# Video Head Impulse Tests with a Remote Camera System: Normative Values of Semicircular Canal Vestibulo-Ocular Reflex Gain in Infants and Children

**DOI:** 10.3389/fneur.2017.00434

**Published:** 2017-09-07

**Authors:** Sylvette R. Wiener-Vacher, Sidney I. Wiener

**Affiliations:** ^1^Vestibulometry Unit, ORL Department, Robert Debré University Hospital, Paris, France; ^2^INSERM U-1141, Robert Debré University Hospital, Paris, France; ^3^Center for Interdisciplinary Research in Biology (CIRB), College de France, CNRS, INSERM, PSL Research University, Paris, France

**Keywords:** balance disorders, vertigo, vestibular, vestibulo-ocular reflex, child development, gaze stabilization

## Abstract

The video head impulse test (VHIT) is widely used to identify semicircular canal function impairments in adults. But classical VHIT testing systems attach goggles tightly to the head, which is not tolerated by infants. Remote video detection of head and eye movements resolves this issue and, here, we report VHIT protocols and normative values for children. Vestibulo-ocular reflex (VOR) gain was measured for all canals of 303 healthy subjects, including 274 children (aged 2.6 months–15 years) and 26 adults (aged 16–67). We used the Synapsys^®^ (Marseilles, France) VHIT Ulmer system whose remote camera measures head and eye movements. HITs were performed at high velocities. Testing typically lasts 5–10 min. In infants as young as 3 months old, VHIT yielded good inter-measure replicability. VOR gain increases rapidly until about the age of 6 years (with variation among canals), then progresses more slowly to reach adult values by the age of 16. Values are more variable among very young children and for the vertical canals, but showed no difference for right versus left head rotations. Normative values of VOR gain are presented to help detect vestibular impairment in patients. VHIT testing prior to cochlear implants could help prevent total vestibular loss and the resulting grave impairments of motor and cognitive development in patients with residual unilateral vestibular function.

## Introduction

An important function of the vestibular system is to stabilize gaze during head movements. Semicircular canal function can be evaluated by measuring the vestibulo-ocular reflex (VOR) to head rotation. The VOR stabilizes gaze by synchronously rotating the eyes in the opposite direction of head movement. The head impulse test (HIT) is a widely used clinical test of the adequacy of the compensatory eye movements in response to application of a sudden, small amplitude head rotation in the plane of each pair of semicircular canals while the patient maintains gaze on a fixed target (1, 2). Severe vestibular canal function loss is characterized by visual observation by the clinician of a corrective saccade sometimes occurring after onset of the head impulse [also referred to as “overt saccades” (1, 3)]. Here, the VOR does not adequately compensate the head movement and the eyes deviate from the target during the rotation. The corrective saccade returns gaze to the target. Note that direct observation of the patient’s eye movements can only diagnose severe loss of canal function—quantitative VOR gain measurements are not possible and corrective saccades cannot always be detected, particularly if they occur early during the head movement.

The use of video recording during head impulse test (VHIT) provides objective and precise measurement of the head velocity during the HIT and more precisely characterizes VOR. It permits calculation of the gain of the VOR, that is, the ratio of the peak velocity of the compensatory eye movement relative to the peak velocity of the applied head movement stimulus. VHIT recording can also reveal corrective saccades occurring during the head movements. These are called “covert saccades” because they are undetectable by visual observation (1). These occur very frequently in cases of mild partial vestibular loss or well compensated vestibular loss (1, 3). Therefore, VHIT is considered as a valuable and valid test for identifying both unilateral and bilateral horizontal and vertical semicircular canal functional loss in adults (2).

For VHIT recordings, adult subjects were equipped with a video camera fixed on goggles tightly attached to the head (2). Results were compared with HIT measured with scleral search coil recordings. The latter is widely considered to be the most precise technique for measuring eye movements. This study showed that VHIT provides reliable data as long as certain parameters are respected. The value of VOR gain decreases with high head velocities particularly above 200°/s and for vertical canal stimulation (2, 3). In cases of vestibular partial loss, the VOR gain may appear to be normal at low head velocities (<100°/s) since compensation processes have taken place, while higher peak head velocities can reveal the canal function loss. Note also that pursuit eye movements are possible at low head velocities (<100°/s) with little or no vestibular participation (4). Thus, the authors proposed that VHIT stimuli should be applied with peak head velocities exceeding 150°/s (2, 3). A more recent study from the same team (5) showed that the gain of the VHIT VOR is influenced by the angle of gaze required for the patient to fixate the target, particularly during vertical canal testing: the gain of the VOR for the vertical canals decreases with the eccentricity of gaze relative to the plane of the vertical canals. A fixation eccentricity of ±40° (corresponding to the canal plane) was thus advised for vertical canal testing (2, 5).

Normal values have recently been published of VOR gain from VHIT tests in healthy adult subjects with goggle-based detection systems (6–8). These studies showed some changes in VOR gain particularly in the aged subjects. But no substantial normative data base of VOR values from HIT is available for children, especially at very young ages, perhaps because of the inadequacy of previously available recording systems and/or protocols. A few publications exist but with smaller samples and older age groups (9–12). Here, we report protocols and results from a recently commercialized VHIT system (13) that records eye and head movements from a distance, without any cumbersome equipment (such as tightly fitting goggles) on the child’s head.

## Materials and Methods

### Subjects

**Figure 1:**
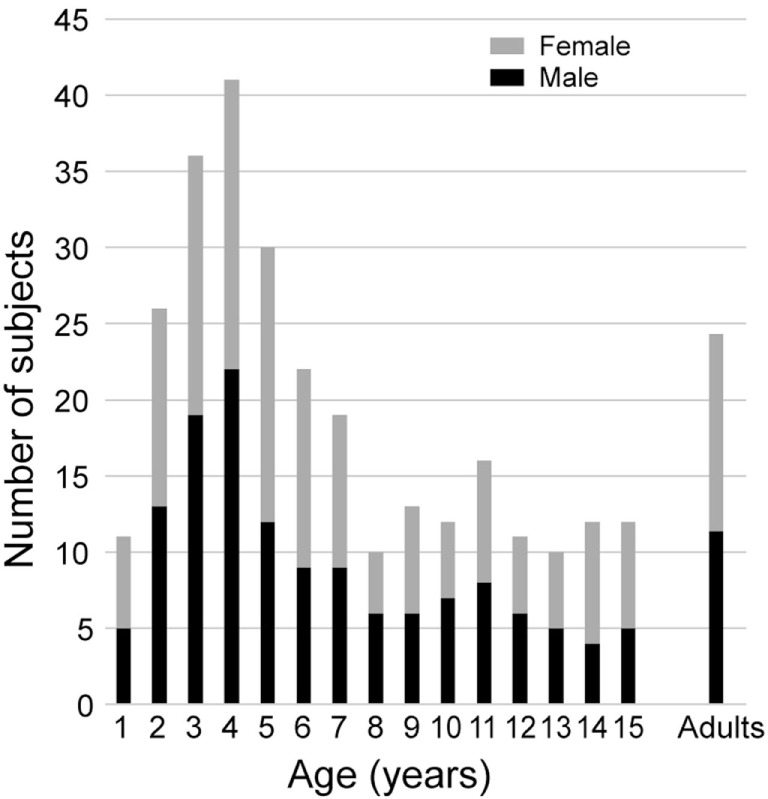
Composition of this pediatric population and adult control group by age and gender.

All subjects were healthy, with no history of neurological, vestibular, ophthalmological, or oculomotor disorders (this was assessed with a complete oto-neuro-vestibular clinical examination and a complete vestibular evaluation test battery) or potentially relevant medical history. Children with corrective eyeglasses were not included. They were recruited from families of hospital staff, their associates, and families of patients. Subjects were selected to provide sufficient samples for 16 age groups that were composed of children aged from 1 to 15 years in 1 year intervals, and an adult group of subjects aged 16 and above for comparison. The population included 274 children with at least 10 subjects per group. Boys and girls were well represented in each group (see Figure 1). The adult group consisted of 26 healthy adolescents and adults (16–67 years old, median = 22.4, 12 males, 14 females). We made efforts to recruit more young children (7 years of age and less) because pilot data showed greater variability than in older children and adults. All subjects were free of any otological, vestibular, or neurological problems. Data are also shown from one 9-year-old child referred for an acute episode of vertigo lasting for several days.

Parents and adult subjects provided written informed consent. Written permission has been received for the use of all identifiable images of children in the figures. The study was conducted in accordance with the ethical standards of the Helsinki Declaration and was approved by the CPP (Comité de Protection des Personnes, Paris, France).

### Setup and Protocol

**Figure 2:**
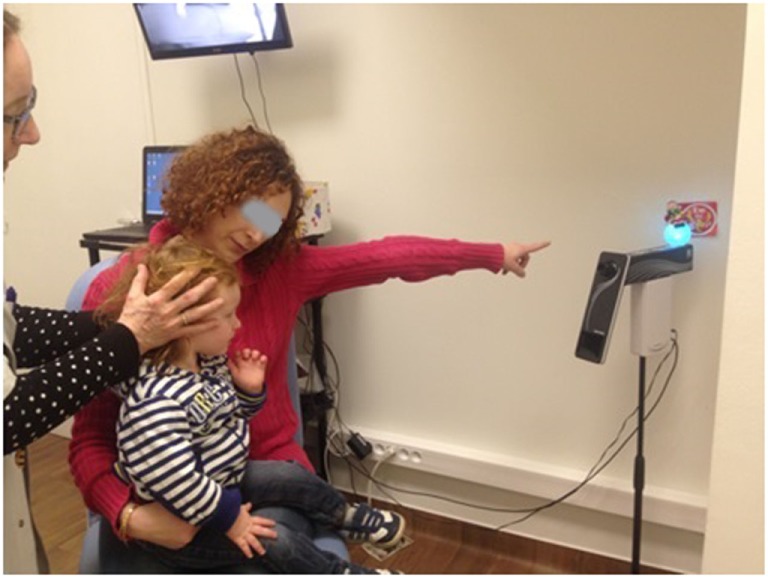
The child (here, aged 18 months) is seated on the lap of his mother while she attracts the child’s attention to a flashing target behind the camera. The operator stands behind the child to rotate the head while consulting the online video display (behind the mother).

**Figure 3:**
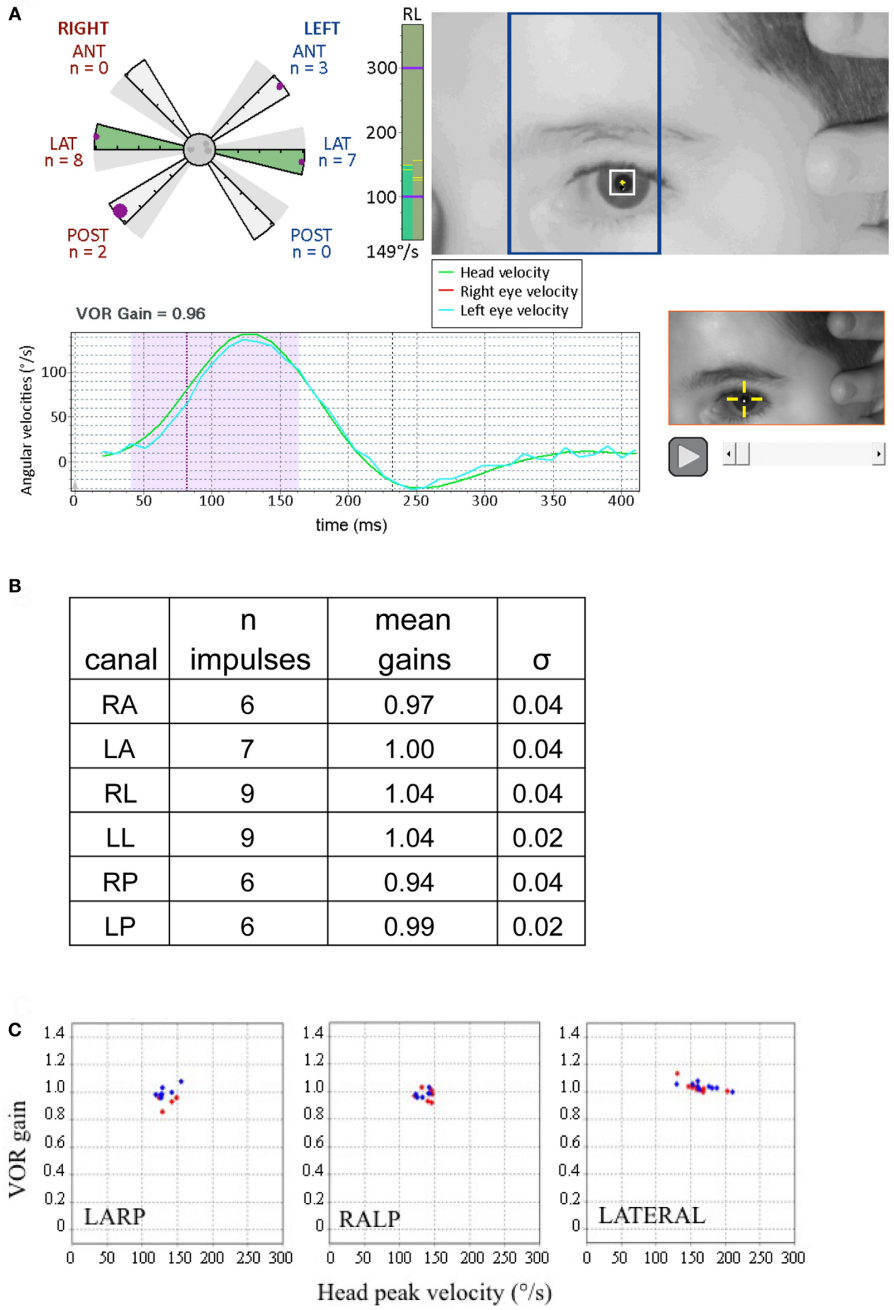
Displays from canal tests of a 9-year-old child with no pathology (adapted from Synapsys^®^ screenshots). **(A)** Impulses of the right posterior canal [in the left anterior and right posterior (LARP) plane]. (*Above right*) On-screen real-time video of the patient’s eye to guide the operator for the upcoming impulse. The blue rectangle indicates the plane to guide the operator to rotate the tilted head in order to optimally stimulate the LARP canals. Before applying the head impulse, the clinician can practice rotating the head slowly while viewing the head and the rectangle to confirm that the head movement is indeed in the correct plane. (*Lower left*) Traces of the head and eye velocities during the most recent impulse performed. The blue trace corresponds to the left eye velocity and the green trace indicates head velocity. The shaded area indicates the data used for gain calculation. (*Upper left*) The polar style plot with six arms (called a “canalogram”) shows the gains of the impulses already executed as filled circles on the shaded slices. The largest circle corresponds to the head impulse most recently performed. The radial length of the canalogram slice corresponds to a gain of one and zero is the edge of the central circle. The gray dots in the central circle correspond to responses set aside by the program (for example, for blinking). In healthy subjects, many gains have the same values and thus some circles may be superimposed. (*Bottom right*) Video recording of the most recent impulse. This can be replayed during testing to help identify the cause of possible artifacts. The vertical green column to the right of the canalogram indicates the velocity of the head for the current head impulse in real time. **(B)** Summary table of results from this subject. **(C)** The vestibulo-ocular reflex (VOR) gains of this examination are plotted as a function of the peak head velocities of the respective impulses. Note the tight clustering, illustrating the good inter-response replicability (this subject’s gain SD’s for respective canals were all inferior to 0.05). Red diamonds correspond to right canal stimulation and blue diamonds to the left (these plots can be displayed on the screen during the test).

The measurements of VOR with the Synapsys^®^ (Marseilles, France) vHIT Ulmer system in children generally followed the same protocol as that for adults (2, 14). Detailed procedures for operating this system have been previously described for adult testing (13). However, some adaptations were required to render the test tolerable for young children. Children under the age of 5 years sit sideways on the lap of a parent or caregiver and face the camera (mounted in front of the child at a distance of 90 cm) (Figure 2). From the age of 5–6 years children are more compliant and can usually sit alone on the chair. The attention of the child is diverted to a small target (a flashing toy, a cell phone, or a tablet screen) directly in front of him or her at a distance from 1 to 1.3 m. The target was affixed to the wall or attached to the back of the camera holder and remained at the same position during the test. Children are asked (or motivated) to maintain their gaze fixed straight ahead on the target. The clinician is positioned behind the child and places her hands on the head of the child (see Figures 2 and 3; Videos S1 and S2 in Supplementary Material). Then the clinician manually applies a brief, unpredictable head turn (the range of angles is approximately 10°–20°) which the subject is instructed not to resist or assist. To apply head impulses in the horizontal plane (for right and left lateral canals), the head is inclined slightly forward at 30° and then rotated right or left. For vertical planes, i.e., for left anterior and right posterior (LARP) and right anterior and left posterior (RALP) canal pairs, the head is positioned at an angle of about 40° to the right or left in the yaw plane relative to the forward fixation point. The head is then rotated forward and back relative to the body [see Figure 1 of Ref. (7)]. Rotations from the left position stimulate the RALP canals while those at the right test the LARP canals. Usually, the lateral canal tests are easier to perform and are tested prior to the vertical ones. The direction (and type of canals) rotations are presented in a sequence as random as possible to avoid anticipatory activity by the subject. During the test the Synapsys^®^ vHIT Ulmer system displays optimal directions and planes of the head rotations for vertical or horizontal canals on the computer monitor (e.g., blue rectangle in Figure 3A, upper right). This is superimposed on the real-time video image of the child’s face during each head movement. This feedback guides the operator to apply the head impulse movements in planes close to those of the pair of canals being tested (see Figures 2 and 3; Video S1 in Supplementary Material). The system automatically sets aside trials outside the desired plane or of insufficient velocity.

### Head and Eye Movement Recording

The video camera of the Synapsys^®^ vHIT Ulmer system has a sampling rate of 100 frames/s. This sampling rate proved sufficient for accurate measurements of the VOR (whose frequency components are far below this sampling rate), for correcting saccades occurring during the VOR as well as for detecting catch-up saccades. Video signals are low pass filtered below 25 Hz by the system, thus avoiding possible aliasing of high-frequency signals. Tests were performed in a well-lit room (to ensure small pupil diameters). Children were seated in an office chair. The height of the chair was adjusted to situate the head at the optimal height for the operator to deliver horizontal or vertical impulses while the subject’s gaze was oriented at the target and centered in the field of the camera.

For each canal plane, the operator tries to rotate the head at a velocity of 120–150°/s (or more; the Synapsys^®^ vHIT Ulmer program can be instructed to set aside trials where head impulses do not reach a desired criterion). The sequence of planes and directions of head impulses can be programmed to be performed randomly, making the head impulse direction as unpredictable as possible to reduce anticipatory activity by the subject (see Video S1 in Supplementary Material). In practice, in older children, we first perform 10 impulses in the horizontal plane, 5 each right and left in random order. Then, we continue with impulses in the respective vertical canal planes (LARP and RALP) testing anterior and posterior canals in random sequence. For very young children, we start with 4–6 lateral canal impulses (right and left) and then we try vertical canal stimulations: a few LARP and a few RALP. If the child is cooperative, we continue with some more lateral canal and vertical canal rotations to obtain at least five validated head impulses in each direction. The chances of succeeding at making a complete test of all canals is improved since the Synapsys^®^ system provides wide flexibility in the sequencing of head impulses permitting shifting from lateral HIT to the two vertical ones and back again within the same session.

The attentive participation of young children must constantly be solicited throughout the tests by using toys, playing diversionary games, offering rewards, and being very patient. In contrast to adults, most children do not anticipate and resist or assist impulses because their attention is diverted by the toy or other target. The absence of neck stiffness makes the head impulses easier to apply. The goal is to apply at least five artifact-free impulses to each of the six canals. The operator should deliver small, passive head rotations, with minimal “bounce-back” (brief counter-rotation) at the end of the head impulse [as previously described for adults by Curthoys et al. (14), McGarvie et al. (7), and Mantokoudis et al. (15)]. Here all tests were performed by the same right-handed experienced operator.

Some children dislike having their head held from behind or being touched by strangers and this is the principal reason of failure of the test in the youngest patients. Having the parent hold the head of the infant firmly in their hands while the operator places her own hand on the top of the parent’s hands to apply impulses sometimes resolves this problem. The criterion of five head impulses at least 150°/s is usually easily achieved for the lateral canals but is sometimes more difficult for the vertical canals (with head velocities more often at 120–140°/s and fewer than five acceptable head impulses). In the youngest patients, we thus sometimes have to be satisfied with velocities as low as 100°/s for the vertical canals since this is the most they will tolerate. In very young children, we always try at the end of the test to apply a few head impulses at velocities higher than 100°/s. If successful, then VOR gains from the session are plotted as a function of head velocity to identify if they are consistent. The entire test typically lasts no longer than 5 or 10 min.

### Data Processing

The system automatically detects the plane in which the head was actually rotated. Impulses are automatically accepted or rejected according to specific criteria (imposed by the software during the test). The criteria for rejection are as follows: the pupil is not detected (inappropriate distance between eye and camera, view out of focus, blinking), the head is not sufficiently stable at the onset of the head impulse, the head rotation amplitude is too small (less than or equal to 7°) even though the acceleration threshold is reached, or the peak acceleration does not reach 2,000°/s^2^ for the horizontal head impulses or 1,500°/s^2^ for the vertical impulses. While records with blinking are initially excluded from the calculation of gain, they are retained for possible later analysis in case some may still permit detection of essential aspects of the eye movement. These are particularly useful in testing infants where there may not be enough impulses without blinks. In our study, the mean gain was calculated on 4–10 responses per subject for each impulse direction.

**Figure 4:**
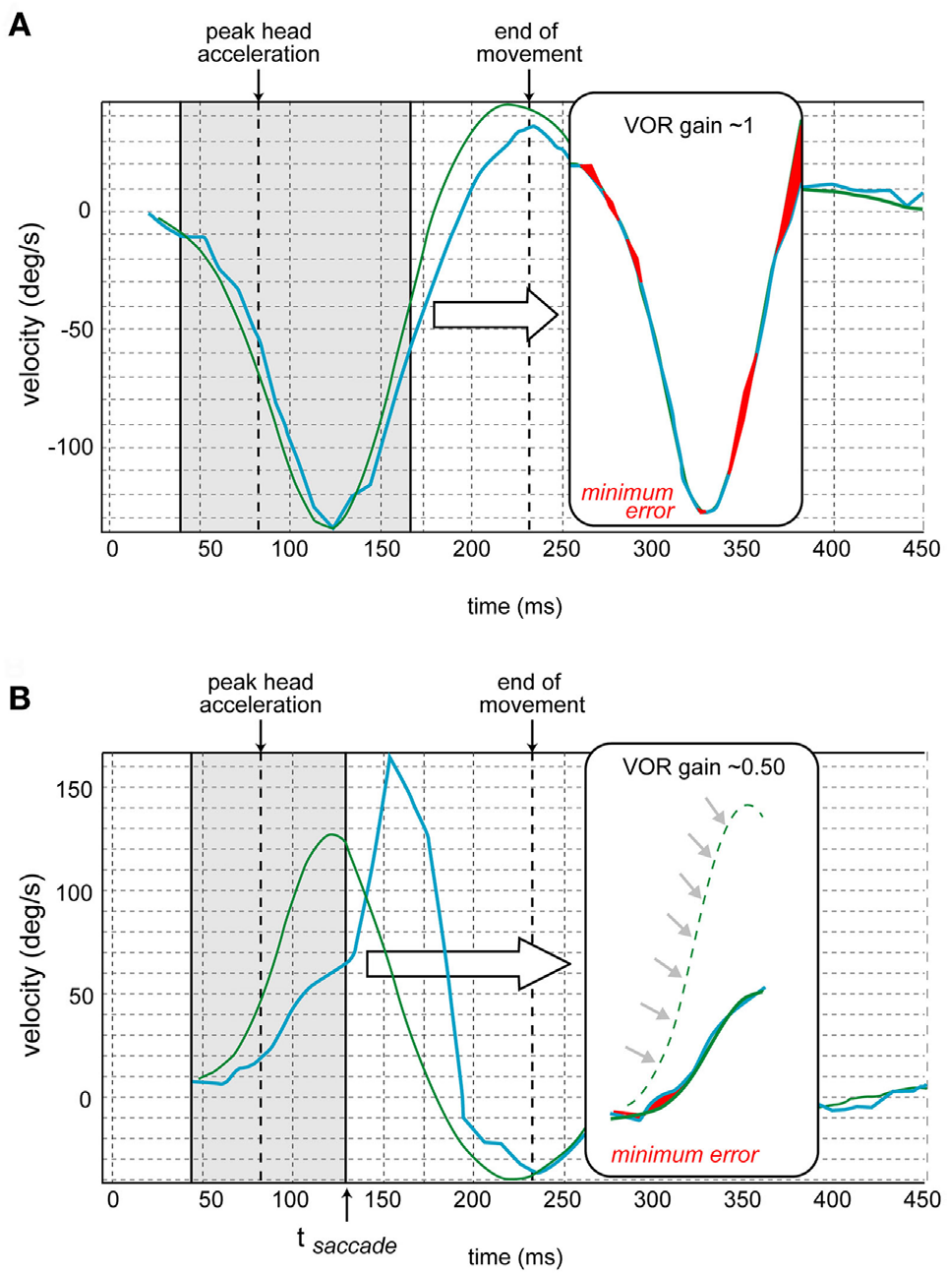
Vestibulo-ocular reflex (VOR) gain calculation [also see Ref. (13)]. **(A)** The traces of the eye movement (in blue) and head movement (in green) velocities are juxtaposed. The program determines the time of the peak head acceleration. Then the gain is calculated over the period from 40 ms before to 80 ms after the peak head acceleration for each impulse. First, the curves of eye and head velocities are aligned by shifted them in time and amplitude to minimize their differences (*via* a least square method by varying two parameters: gain and latency). The red shaded areas in the insets correspond to this minimized error. The resulting aligned traces appear in the inset. **(B)** In case of a corrective saccade (not occurring in normal subjects but characteristic of pathological cases), the same calculation is performed but the period analyzed (in brackets) ends at the time of the onset of the saccade (*t*_saccade_) that corresponds to the inflection point of the eye velocity. The resulting aligned traces appear in the inset. This schematic is from a different canal where the velocity is opposite that of Figure 4A (adapted from images provided by Synapsys^®^).

Prior to calculating gain, each individual head and eye velocity record is examined by the clinician to ensure that an acceptable stimulus and recording were achieved, free of any artifacts (e.g., blinking, bounce-backs, change of gaze during the head movement due to loss of attention to the target). Individual video recordings can also be replayed prior to gain calculation. VOR mean gain and peak head velocity were calculated for each subject and evaluated for each of the 16 age groups. The VOR gain calculation is explained in Figure 4 (13). Briefly, the head and eye velocity curves are aligned *via* a least-squares best fit, and the parameters required for the optimal alignment are the basis of the gain calculation. The program detects onset of VOR in the first 18 ms after the beginning of the head movement (this is the range of VOR latency in normal subjects). If the latency is greater than 18 ms (only found in subjects with neuropathies and neurological disorders) the trace before the VOR will be included in the gain calculation, underestimating the real gain of the delayed VOR (and this will have to be corrected, taking in account the latency). Corrective saccades (characteristic of pathological cases) are identified with an algorithm that detects deviations of the orientation of the eye of amplitudes greater or equal to 2° relative to the target. The algorithm also computes when saccades occur by detecting the inflection point of the eye velocity. Saccade timing is obtained from the peak angular acceleration of the eye (Figure 4B). In the case of a corrective saccade occurring during the head movement (covert saccade), only the eye movements prior to this saccade are used for VOR calculation.

### Analyses and Statistics

The asymmetry was calculated for each canal type (anterior, lateral, or posterior) as the absolute value of the difference between the left and right gains. We used the Sigmaplot^®^ (Systat Software Inc.) for statistical tests. These included one-way ANOVA, Dunn’s *post hoc* multiple comparison test, Mann–Whitney rank sum test, and Pearson moment product correlation. Separate ANOVAs were carried out for the respective canals and confidence intervals were calculated from the subjects’ mean VOR gains, taking in account the size of each age sample (with 10 or more subjects per age group). The 95% confidence intervals were calculated as *Z* values of 1.96 times the SEM using an online tool (16). The CONSUME analysis (17) was implemented as a formula in Microsoft^®^ Excel to determine inflection points in the plots of VOR gain as a function of age. All individual data values from each canal (left and right) were used for this.

## Results

**Figure 5:**
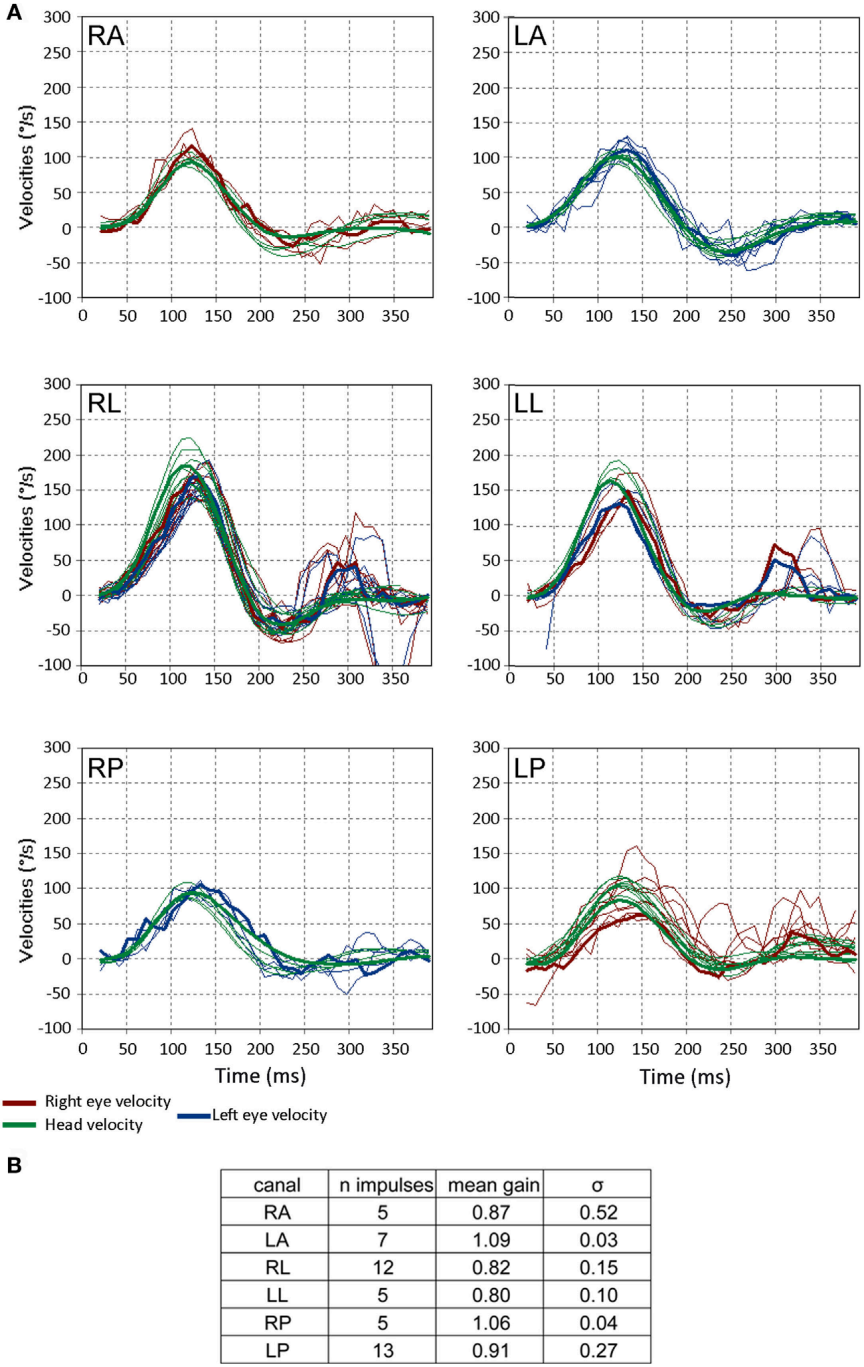
Video head impulse test (VHIT) responses from a 3-month-old child with no pathology. **(A)** The format is the same as in Figure 3. Note that a few saccades occur more than 200 ms after the head impulse is finished and these correspond to change in gaze, not corrective saccades. These reflect the instability of gaze in small children and are unrelated to the vestibulo-ocular reflex. **(B)** Gains and their SDs (σ) for the respective canals for this subject. Note the values of σ of the gain are greater than those of the older child in Figure 3C (figures adapted from Synapsys^®^ VHIT Ulmer program screenshots).

**Figure 6:**
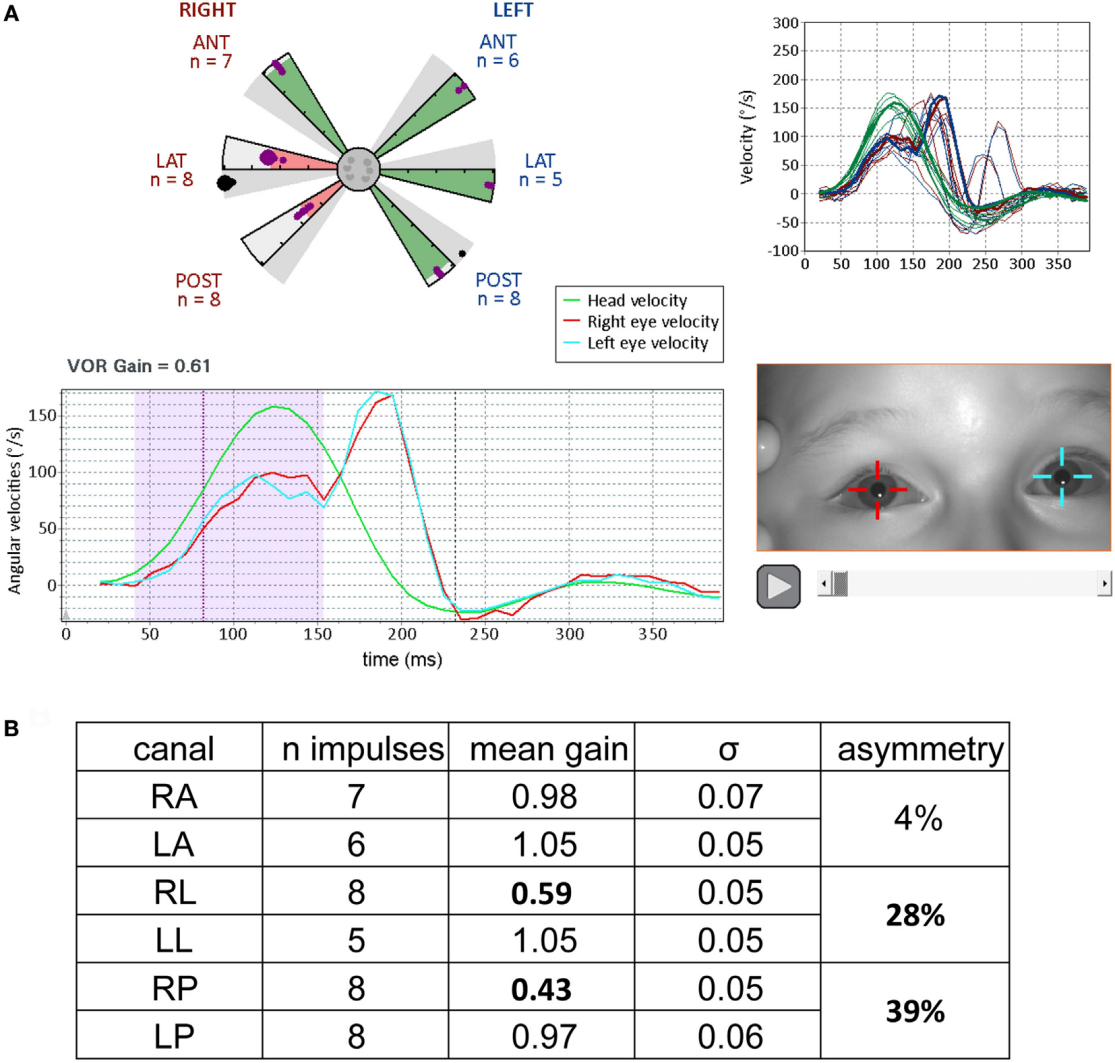
Results from video head impulse test (VHIT) performed on a 9-year-old child referred for an acute episode of vertigo lasting for several days (same formats as Figures 3 and 5, with analysis of the upper part of Figure 4). Partial vestibular loss in the right lateral and posterior canals is indicated by abnormally low gains in the corresponding (pink shaded) arms of the canalograms while gain is normal in the other four canals (green shaded arms of the canalogram). The dots appearing in the gray shaded slices of the canalograms indicate as a gain value the efficiency of the first corrective saccade to shift gaze back to the target. **(A)** Lower left, for a right lateral head impulse, the peak of the left eye movement trace (blue) is at a lower velocity relative to the head movement trace (green)—this corresponds to a reduction in gain. The corrective saccade starts at about 150 ms. Upper right, overlays of traces from all right lateral head impulses performed. **(B)** Summary table of results. The gain values for the pathological measures show low variability similar to the measures in normal subjects (figures adapted from Synapsys^®^ vHIT Ulmer data processing program screenshots).

Performing the VHIT for vertical canals is more challenging with very young children. Both lateral canals were successfully tested in all children but, in a few (*n* = 10) children, all younger than 3 years old, all vertical canal rotations could not be replicated at least five times at high velocities. However, complete tests were possible in infants as young as 3 months of age (see Figure 5). As in this example, in the youngest infants vertical head rotations were only 100°/s because higher velocities were not tolerated. In total, 548 lateral canals, 536 anterior canals, and 537 posterior canals were tested in this healthy population. We regularly perform this protocol in children showing symptoms of vestibular impairment, and it reveals pathological responses (Figure 6 provides an example).

**Figure 7:**
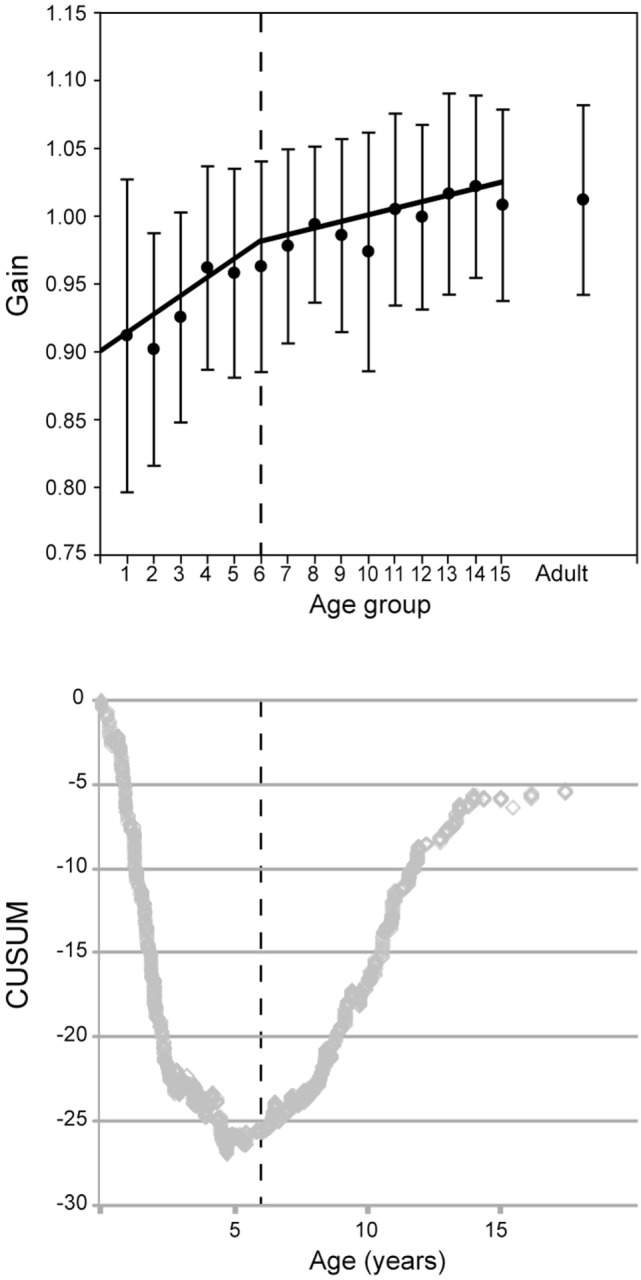
Vestibulo-ocular reflex gain evolution with age for all canals combined. (Above) mean gain (circles) and SD (error bars) with separate regressions for children ages up to 6 (groups 1–5) and 6 and above (groups 6–15). (Below) The CUSUM analysis shows a major inflection point at about age 6 (vertical dashed lines).

In data from all canal stimulations pooled together, the values of VOR gain increase significantly with age (analysis for age groups 1 through 15; Pearson Product Moment correlation coefficent = 0.39, *p* < 0.001) (Figure 7). A Kruskal–Wallis test of gain values also showed variation with age (*H* = 89.7, df = 15, *p* < 0.001; non-parametric statistics were used since this data set did not pass the Shapiro–Wilk normality test, *p* > 0.05). Dunn’s multiple comparison *post hoc* tests (*p* < 0.001) revealed that gain values for the age groups of 1, 2, and 3 years were significantly lower than those of children in age groups 10 through 15 and adults. No statistically significant difference was found between children aged 10–15 years and adults (Dunn’s multiple comparison *post hoc* tests, *p* > 0.05). The normative values of these data, with 95% confidence limits appear in Table 1.

**Table 1:** Normative values of vestibulo-ocular reflex gain as a function of age (Cl = Confidence limit).

Age group	Anterior canal			Lateral canal			Posterior canal		
	Mean gain	95% CI lower	95% CI upper	Mean gain	95% CI lower	95% CI upper	Mean gain	95% CI lower	95% CI upper
1	0.95	0.88	1.02	0.90	0.85	0.97	0.87	0.82	0.92
2	0.95	0.92	0.98	0.88	0.86	0.90	0.89	0.86	0.92
3	0.95	0.93	0.97	0.91	0.89	0.92	0.93	0.91	0.95
4	0.98	0.96	0.99	0.95	0.93	0.96	0.96	0.94	0.97
5	0.97	0.95	0.99	0.95	0.93	0.97	0.96	0.94	0.98
6	0.98	0.96	1.00	0.96	0.94	0.98	0.94	0.92	0.96
7	0.98	0.96	1.00	0.97	0.95	0.99	0.96	0.94	0.98
8	1.02	1.00	1.04	0.98	0.95	1.00	0.98	0.96	1.00
9	1.00	0.99	1.01	0.98	0.94	1.01	0.98	0.96	1.00
10	0.99	0.97	1.02	0.98	0.95	1.01	0.98	0.96	1.00
11	1.00	0.98	1.02	1.01	0.98	1.03	1.00	0.97	1.02
12	1.02	0.98	1.05	0.99	0.96	1.01	0.99	0.96	1.02
13	1.02	0.98	1.05	1.00	0.98	1.02	1.03	0.99	1.06
14	1.03	1.00	1.06	1.01	0.98	1.04	1.02	0.99	1.05
15	1.02	0.99	1.05	1.02	1.00	1.04	0.99	0.96	1.02
Adult	1.03	1.01	1.05	1.02	1.00	1.04	0.99	0.97	1.01

However, the evolution of the VOR gain with age does not seem to be a simple linear progression. To reveal inflection point(s), we employed the CUSUM method (17). Changes of slope of the CUSUM curve indicate inflection points in the original data. This shows a major inflection point at 6 years of age and another at age 16 (see Figure 7). For this reason, the data in Figure 6 are plotted as two regressions for data before and after age 6, respectively. In Figure 7, groups 1–6 give a Pearson moment correlation coefficient of 0.25 (*p* < 0.01) and for groups 7–15, the Pearson moment correlation coefficient is 0.10 (*p* < 0.01).

Successive measures from the same subject showed low variability (for example, Figure 3B). For each subject, the SD (σ) was calculated for the measures for each of the six canals (e.g., Figures 5B and 6B). These were then averaged together for each age group and the values are presented in Table 2. The intra-individual variations for the VOR gain measures from the respective canals appear to decrease with age until about the age of 6 or 7, then remain relatively stable.

**Table 2:** Mean sigma values of the intra-individual variability of gains.

Age group	Mean σ
1	0.106
2	0.102
3	0.107
4	0.098
5	0.095
6	0.084
7	0.082
8	0.061
9	0.069
10	0.080
11	0.059
12	0.068
13	0.057
14	0.057
15	0.059
Adult	0.066

In this normal pediatric population, we did not find any statistically significant difference between gains on the right or left side for lateral, anterior, or posterior canals (Mann–Whitney rank sum test, *p* > 0.05). The gain asymmetry between right and left stimulation (expressed as percentages with zero as no asymmetry) was 4 ± 1% for the lateral canals, 6 ± 4% for the anterior canals, and 6 ± 2% for the posterior canals. For children younger than 2 years and only for anterior canals, the asymmetry was greater (15 ± 7%) (see Table 3). Thus, the data from the corresponding canals on the left and right sides are combined in the following analyses.

**Table 3:** Asymmetry values as a function of age and type of canal.

Age group	Anterior canals		Lateral canals		Posterior canals	
	Asymmetry	SD	Asymmetry	SD	Asymmetry	SD
1	0.15	0.08	0.04	0.03	0.06	0.06
2	0.15	0.06	0.05	0.04	0.09	0.07
3	0.06	0.05	0.05	0.04	0.09	0.07
4	0.07	0.06	0.04	0.04	0.08	0.07
5	0.05	0.03	0.04	0.04	0.08	0.05
6	0.06	0.05	0.04	0.04	0.06	0.06
7	0.05	0.06	0.04	0.03	0.04	0.03
8	0.05	0.04	0.03	0.03	0.06	0.06
9	0.06	0.05	0.02	0.03	0.05	0.02
10	0.08	0.07	0.04	0.04	0.08	0.09
11	0.05	0.04	0.04	0.03	0.06	0.04
12	0.05	0.03	0.03	0.03	0.04	0.04
13	0.03	0.02	0.04	0.04	0.05	0.03
14	0.05	0.05	0.03	0.03	0.03	0.02
15	0.07	0.09	0.02	0.01	0.06	0.07
Adult	0.06	0.04	0.04	0.04	0.05	0.05

Comparisons of VOR gain for the respective canals and with age showed that both of these factors are significant (two-way ANOVA: age df = 15, *p* < 0.001; canals df = 2, *p* < 0.001; interaction not significant: df = 30, *p* = 0.127). Values for the anterior canals were on average 3% greater than those of the lateral and posterior canals (Holm–Sidak method *p* < 0.001), which were not significantly different from one another (*p* = 0.513). For this reason, further analyses were pursued on the three respective canal types separately.

**Figure 8:**
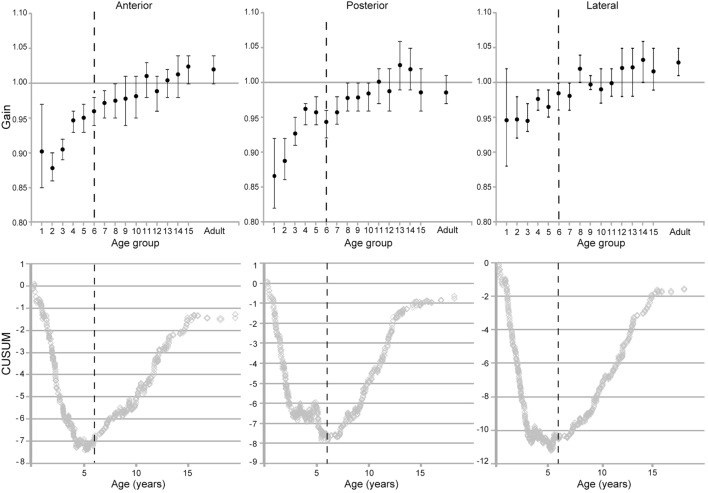
Evolution of vestibulo-ocular reflex gain with age for the respective canals (means and two-tailed 95% confidence intervals). (Below) CUSUM analyses of the raw data from the respective plots.

The individual canals showed similar patterns of increase in VOR gain with age (Figure 8). Calculation of confidence intervals around the means of the VOR gain as a function of age show that the value of 1 was within the 95% confidence interval only for children older than 10 years old for lateral and posterior canals and older than 9 years old for the anterior canals.

To more clearly define these effects, possible gender differences in the evolution of gain with age were examined for the respective canals. The data for each of the canals were normally distributed (Shapiro–Wilks test, *p* < 0.05). This permitted two-way ANOVAs that showed significant effects of the age factor for all canals (*p* < 0.001). A significant effect of gender appeared for the anterior canals only (*p* = 0.046) with values of 1.001 ± 0.005 for females and 0.986 ± 0.005 for males. This difference is minor relative to the age differences. Significant interaction factors were found for age and gender for the lateral canals only. Pairwise multiple comparisons showed differences between girls and boys in age groups 1, 4, and 9 (Holm–Sidak method, *p* < 0.05). In group 1, this can be accounted for by the variations in ages of the samples of boys and girls for this period of intense development, while in group 4, the mean difference in gain was only 3.2%. In group 9, the gain for boys was 0.93 ± 0.02 while for girls it was 1.05 ± 0.02.

The CUSUM analyses of data from the individual canals also reveal two inflection points. The first one was at 69.5 months for the lateral canals, 68–72 months for the posterior canals and 68 months for the anterior canals, and thus these are fairly consistent. A second inflection point appeared at age 16 for each canal type. As with the grouped data, there is a more rapid increase in VOR gain from groups 1–6. A slower increase in groups 7 through 15 and, finally, a plateau at adult levels.

Inter-individual variability of the gain measurement is low (mean of the SDs = 0.09 for the children groups and 0.06 for the adult group; see error bars on Figure 7). This variability decreases overall with age from group 1–15 (Pearson product moment correlation coefficient *r* = −0.159, *p* < 0.001) and also in groups 1 through 6 (Pearson product moment correlation coefficient *r* = −0.0805, *p* = 0.0154). However, in groups 7 through 15, no significant difference was found (Pearson product moment correlation coefficient *r* = −0.00150, *p* = 0.969).

## Discussion

### Methodological Considerations

Video head impulse test is a valid, practical, and rapid diagnostic tool for detecting vestibular deficits in children as young as 3 months of age. Unlike HIT performed only by visual observation by a clinician, video measurement permits detection of covert corrective saccades, indicators of a severe deficit. A further advantage of VHIT is its utility in detecting partial loss when VOR gains are on the order of 0.6 or 0.7 in the absence or presence of irregularly occurring covert saccades. While VHIT has been convincingly validated in adolescents and adults by comparison with scleral search coil measurements (2), goggle-mounted video systems have proven impractical for infants who do not tolerate it. However, the use of remote video detection and specific adaptations of the protocol (described here) have now rendered this powerful technique practical for pediatric testing. The low variability of repeated measures to the same stimulus in these young subjects attests to the reliability of the measurements. Furthermore, the test typically lasts only 5–10 min. However, it is essential that the clinician performing the rotations be well experienced. A system of video feedback guiding the operator for the planes of rotation (as provided by the Synapsys^®^ system) is particularly helpful for this training.

However, the vHIT approach has limitations in children, particularly the youngest. They do not always accept having someone rotate their head. Furthermore, the attention span is often short in young children and their gaze wanders from the target. In order to motivate the young children to keep looking at the target, we create diversions (such as a laser pointer flashed on the target, flashing toys, cell phone displays, or video tablets). But these targets can be larger than those used in older children and adults, and this could increase the variability of the VOR measurements in these very young children (as we observed). However, Irving et al. (18) observed that the accuracy of the saccades improves in young children with larger targets (as opposed to small dots) by more effectively attracting their attention. In very young children, the distance from the eye to the fixation target is also more difficult to control. But here it was always maintained in a range from 100 to 130 cm, thus not requiring convergence, which could be a source of variability for the measurements (19).

We did not find any statistically significant differences between right and left VOR gains for horizontal, anterior, and lateral canals in these normal children. However, significant differences between the VOR gains for rightward or leftward head rotations were reported in adults (7) with the goggles-mounted video recording system. The present results support those authors’ suggestion that this difference was related to their system whose measures are limited to monocular recordings of right eye movements only (3). Note that the Synapsys^®^ Ulmer remote video system also records only one eye for vertical stimulations, but it is always ipsilateral to the canal being tested. This is likely to reduce the asymmetry detected. Remote video recording also eliminates the problem of artifacts due to the slippage of the goggles on the head during rotation, permitting the classic analysis of head and eye velocity rather than position (discussed below). There is also no need for calibration since the eye and head movement are recorded on the same image.

As mentioned in the Introduction, Weber et al. (3) and MacDougall et al. (2) counsel that VHIT should be performed at high head velocities in clinical testing. Impulses with low peak head velocity (below 100°/s) are ineffective in revealing partial loss of canal function. Most patients with unilateral vestibular loss can generate VOR gains in the normal range at these low velocities (2, 3), likely because these velocities are commonly experienced in everyday life permitting compensation processes to occur (2). It is rather easy to apply head rotations greater than or equal to 150°/s to the lateral canals in children because of their low rigidity of the neck. For the vertical canals, it was difficult to reach 150°/s and often the head speed was only 120–140°/s, and as low as 100°/s for the youngest.

The very lowest speeds may not be a serious problem because in very young infants smooth pursuit is not “mature” (i.e., near adult performance levels) until the age of 5–6 months (20). In adults, rapid head velocities are required because at head velocities ≤100°/s, smooth pursuit can compensate for pathologically low VOR gain. It is possible then that at ages less than 5–6 months smooth pursuit is not mature enough to compensate reduced VOR gain and, thus, at those ages slower head velocity stimuli in VHIT could reveal diminished VOR gains. In infants tested at 100°/s, it is possible to detect asymmetry between the responses on the two sides and this could also indicate hypofunction.

### Normative Values of VOR Gain and Clinical Application

This study brings new perspectives to previous work on normal adult subjects (6–8, 13) or with much smaller populations of children older than 3 years (10–12). Complementing these studies, we provide here normative data for gains in children. The 95% confidence limits indicate reference values for comparison of results from clinical patients. Abnormal values (in general <0.80) should be compared to the results of a full vestibular test battery, including oculomotor, neurological, and vestibular testing with caloric, rotatory chair testing, and cervical vestibular evoked myogenic potential (Cvemp) before conclusions are drawn concerning pathology. Because of the difficulties testing children 2 years old and younger and the greater variability at these ages, a second confirmatory test is advised in case of abnormally low gain values. In general, gains lower than normal indicate a vestibular loss (2, 3, 14, 21). However, VOR gains above than the normal range occur when abnormal saccades overshoot the target and this could correspond to cerebellar disorders (22–24).

McGarvie et al. (7) published normative values of VOR gain in goggle-based video measurements of the right eye during VHIT tests of the respective canals in adults. Note that in contrast with classical velocity measurements (used here), they calculated gain from position data since they found that this better resisted artifacts due to goggle slippage. They report that gains remained relatively constant up to the age of 70 and, thus, we combined the data of our adult group aged 16–67. However, in our pediatric population, the gain values increased with age and leveled out to adult values by age 16. Note that our confidence limits of gains for the respective canals include the value of 1.0 in our older children and adults, but this is outside or at the upper limit of the range for the left posterior stimulations for adults in the McGarvie et al.’s (7) work. These authors explain that their values are likely affected by measurements only being made from the right eye.

The VOR gains of the anterior canals had consistently higher gain values (albeit by only 3%) than the posterior or horizontal canals at all ages. To our knowledge, this is the first such observation in the literature, and further investigation will be required before proposing an explanation.

### Non-Linear Evolution of VOR Gain with Age

Here, the youngest age groups had lower values of VOR gain and this evolved monotonically but irregularly with age. The CUSUM analysis revealed an inflection point at the age of 6 years (appearing at 4–5 years for the anterior canals and six for the lateral and posterior canals). From then on gains increased more gradually with age until leveling off to adult levels at age 16. The rapid changes in VOR gain for high head velocity impulses during the first 5–6 years of life could be related to anatomical development and/or maturation of central pathways since the vestibular end organ and myelination of the first order vestibular afferents are reported to be mature at or shortly after birth [e.g., Ref. (25)].

Anatomical development may play a role in these variations in the rate of evolution of VOR gain. Note that inflection points also appear around the age of 6 for the development of skull size (26) and interpupillary distance (IPD) (27). Thus, the vestibulo-oculomotor circuitry must be revised with growth of the skull and concomitant changes in the IPD, the distance separating the vestibules and musculoskeletal changes. Variation of IPD with age could modify resting vergence angle and, thus, the VOR gain. But based on the IPD in a large cohort from infancy to 19 years (27), the variation of vergence with age was negligible for the distances (100–130 cm) employed here (3/10 of a degree over 15 years). Thus, changes in vergence requirements are not considered to have an impact the changes in evolution of gain with age.

Adaptive circuitry changes might be reflected in VOR gain evolution with age. The data of Irving et al. (28) indicate that asymptotic peak velocity for saccades increases from 3 to 6 years of age, then levels off until the ages of 11 and 12. Note, however, that for cervical Vestibular Evoked Myogenic Potential (cVEMP), the principal inflection point appears at the age of 11–12 years (Wiener-Vacher and Wiener, unpublished observations). This suggests that the change in rate of VOR gain increase at 6 years of age is selective for oculomotor but not neck muscle responses to vestibular stimulation. However, published data on the influence of vestibular signals on the maintenance of postural balance as measured by the Sensory Organization Test (SOT) (29) also appear to show the same transition as shown here for the VHIT VOR under the condition where only vestibular (but not visual or somatosensory) inputs are stable: consistent with this, the SOT data of Figure 2 of Steindl et al. (30) appears to have inflection points at both 6 and 16 years of age. Studies with low velocity head movements in young animal models (31) and infants (32, 33) showed that VOR gains are much lower than those in adults and increase progressively during development. These results are not comparable with the present ones because low velocity VOR engages different mechanisms. suggested to indicate a delay in maturation of the cerebellar control of the VOR (22, 31–33). The low VOR gains observed here in children younger than 4–6 years could correspond to immaturity of the cerebellar control on VOR and the rapid increase of the gains during the 6 first years of life may correspond to the time necessary for this control to take place.

Refraction characteristics vary during childhood and could impair viewing the target during VHIT and introduce variability in VOR gain. Near vision in children (at a distance of 40 cm) is not optimal (34, 35). The hypermetropia that is observed in normal newborns decreases during the first 3–4 years of life to reach normal level of refraction (emmetropia, as found in adults) by the ages of 6–8 years (34) meaning that near vision is not as precise in very young children. Since the visual system is required for the calibration of the VOR (32), it is possible that, in very young children, the innate hypermetropic vision may contribute to the variability of the VOR gain observed in age groups 1–3.

### Clinical Perspectives

The availability of a sensitive, rapid, and reliable test of vestibular function has an important potential clinical impact. Many children are referred for vertigo symptoms and too often are subjected to unnecessary and costly brain imaging procedures (requiring general anesthesia in infants) which could be avoided by a rapid VHIT screening and, eventually, a subsequent battery of vestibular tests. Furthermore, it is imperative that vestibular loss be detected as early as possible. In cases of profound vestibular loss, adapted rehabilitative therapy should begin promptly, including the active participation of the family and appropriate modifications of the home environment (36). Untreated profound vestibular loss leads to dangerous instability, visual impairments, and delays as well as possible deficits in cognitive development [(37, 38); also see Ref. (39)]. Interestingly, during normal development children acquire the capacity to orient their movements relative to configurations of environmental cues (termed “place navigation”), from the ages of 6–7 (40), coinciding with stabilization of VOR gain evolution observed here. Such development issues can be of particular concern in young children passing through critical periods of development since vestibular impairments are frequently comorbid with hearing loss. Thus, prior to cochlear implantation of auditory prostheses, it is vital to determine if only one vestibule maintains residual function in order to make every effort to preserve this essential sensory input by performing only one implant at a time (41). Since these implants are now done in very young children, VHIT is a practical choice for such screening.

## Conclusion

Remote video recordings and adapted protocols permit HIT in children as young as 3 months old in less than 10 min. The normative values of VOR gain presented here can serve as a reference for comparison of results from pediatric patients of different ages. While VOR gain is reported to remain relatively constant after the age of 16 (5, 7), there is a rapid increase until about the age of 6, following by a more moderate evolution to adult levels.

## Ethics Statement

Parents and adult subjects provided written informed consent. The study was conducted in accordance with the ethical standards of the Helsinki Declaration and was approved by the CPP (Comité de Protection des Personnes, Paris, France).

## Author Contributions

SW-V conceived of the experimental design, carried out the experiments, analyzed and interpreted the data, prepared figures, and drafted and revised the paper. SW made substantial contributions to analysis, interpretation, and presentation of the data, and revised the manuscript critically for important intellectual content. Both authors gave final approval of the version to be published and agree to be accountable for all aspects of the work in ensuring that questions related to the accuracy or integrity of any part of the work are appropriately investigated and resolved.

## Conflict of Interest Statement

The authors have no conflict of interest to declare. Synapsys^®^ provided images and video recordings and helped to defray costs for presentation of some of these results at scientific congresses.

## Supplementary Material

Video S1 vHIT testing of a 9 year old child with screenshots from the Synapsys^®^ (Marseilles, France) VHIT Ulmer system.

Video S2 vHIT testing of a 10 month old child with the Synapsys^®^ (Marseilles, France) VHIT Ulmer system.
